# Maintaining hope after a disabling stroke: A longitudinal qualitative study of patients’ experiences, views, information needs and approaches towards making treatment decisions

**DOI:** 10.1371/journal.pone.0222500

**Published:** 2019-09-13

**Authors:** Akila Visvanathan, Gillian Mead, Martin Dennis, William Whiteley, Fergus Doubal, Julia Lawton

**Affiliations:** 1 Centre for Clinical Brain Sciences, The University of Edinburgh, Edinburgh, United Kingdom; 2 Usher Institute of Population Health Sciences and Informatics, The University of Edinburgh, Edinburgh, United Kingdom; University of Brighton, UNITED KINGDOM

## Abstract

**Background:**

Some treatments after a disabling stroke increase the likelihood patients will survive longer but with significant disability. Patients and doctors should make collaborative decisions regarding these treatments. However, this can be challenging. To better understand treatment decision-making in acute disabling stroke, we explored the experiences, views and needs of stroke survivors in hospital and six months later.

**Methods:**

Fifteen patients who had a disabling stroke were interviewed within a week of their diagnosis; eleven were re-interviewed six months later. Data were analysed thematically and longitudinally.

**Results:**

Patients’ functional abilities prior to their stroke and need for hope of functional recovery appeared to impact on their involvement in decision-making. In the early period post stroke, patients who were functionally independent pre- stroke described being emotionally devastated and ill-prepared for the consequences of stroke. They appeared unaware that treatments offered might extend their life but with significant disability and took all treatments in the hope of functional recovery. Those who were dependent pre-stroke appeared to be more stoic, had considered treatment implications and decided against such treatments. At follow-up, all patients had varying unmet psychological needs which appeared to contribute to poor quality of life. In the early period post stroke, patients looked for various ways to cultivate and maintain hope of functional recovery. While patients continued to look for hope at six months, they also reported wishing they had been given realistic information in the early period after stroke in order to prepare for the consequences.

**Conclusion:**

Stroke survivors may benefit from psychological support. A collaborative approach towards treatment decision-making may not be realistic in all patients especially when they may be emotionally distressed and looking to maintain a positive outlook. Communication strategies to balance maintaining hope without providing false hope may be appropriate. Patients’ information needs may need reassessed at different time points.

## Introduction

Each year approximately 150,000 people have a stroke in the UK. [1] In the UK, stroke is the leading cause of reported severe disability. [2] Almost two-thirds of stroke survivors leave hospital with a disability. [3] More than half are left dependent on others for everyday activities and may require long-term, institutional care (e.g., in hospital or a care home). [3,4] Therefore, suffering a stroke can be life-changing and stroke patients need to access appropriate treatment during the acute phase as well as follow-on rehabilitation.

In the UK, acute treatment of stroke and follow-on rehabilitation has improved in recent years. [5,6] Relative to 20 years ago, patients generally have better access to treatment and care. [7] Yet there remain significant challenges with respect to stroke care. Notably, there are different options for treatments during the acute phase, which are each associated with different outcomes. Some treatments (e.g. thrombolysis [8] and mechanical thrombectomy[9]) improve functional outcomes for patients who survive the stroke (i.e. it is less likely for patients to be left with significant physical disability as a result of these treatments). However, other treatments (e.g., intermittent pneumatic compression for prevention of deep venous thrombosis, [10] antibiotics for treating infections, [11] parenteral fluids [12] and enteral feeding [feeding through a tube placed into the stomach through the nose or surgically into the abdomen] [13]) increase the likelihood that patients who have suffered a severe stroke and been left with significant disability will survive longer. Therefore, the net effect is that more patients will survive with significant disability as a result of these latter treatments.

Therefore, a key decision that patients, along with their doctors need to make is: should the patient receive treatment that increases the likelihood that s/he will survive the stroke but with a significant disability *or* forego such treatments and, in turn, accept the increased risk that s/he will die. This is a difficult question and there are no easy answers. Making decisions regarding stroke treatments typically warrants careful discussion between the patient, their family, their doctor and the multidisciplinary team–a “shared decision-making” process. [14] Yet, in practice, (particularly, in the early period after an acute disabling stroke) implementing effective shared decision-making can be challenging. [15] This issue has been under-studied with respect to acute stroke. Exploring these challenges goes to the heart of the issues considered in this paper.

Firstly, acutely unwell patients may be in shock as result of having been given a life-changing diagnosis. Literature from critically unwell patients [16] including in coronary care [17] have highlighted how a sudden deterioration in health may leave patients fearful and distressed; [18] especially when they have not considered a situation where they may be left disabled. Critically ill patients, such as those who have suffered a severe stroke, may lack capacity (that is, lack the ability to understand, process and weigh up information to make a decision) [17] and their inability to engage with health professionals may make them ineffective partners in a shared decision-making process. [18] However, much of the research around this issue has focused on the experiences of patients with chronic, progressive conditions such as dementia [19] (rather than stroke) and how these patients engage with health professionals to make treatment decisions. The experiences and reactions to diagnosis of those who have suffered an acute disabling stroke are under-reported–we go on to explore this key area in this paper.

Secondly, an important step in shared decision-making is for health professionals to provide necessary information to patients. This is intended to enable patients to arrive at an appropriate treatment decision having properly weighed the outcome-related risks, such as those outlined above. In conditions such as cancer and dementia, providing information to patients has been shown to help them understand their diagnosis and make decisions regarding treatments. [20–22]Decision aids (such as leaflets and educational programmes) in stroke have been reported to help patients understand their diagnosis. However, most of these aids have been tested just before patients are discharged from hospital or in the outpatient setting. Moreover, those severely affected by the stroke had been excluded from these studies. [23] Hence, the transferability of these interventions to an acute setting and to patients severely disabled as a result of their stroke is uncertain. While guidelines published by professional bodies such as the General Medical council,[24] American Heart Association [25] and Royal College of Physicians [26] iterate the need for early, timely and tailored information delivery to patients, we need to consider how this recommendation may affect those who have had a severe stroke who, like many critically unwell patients, may not be able to fully process this information. [17] Overall, the information needs of acute disabling stroke patients to make decisions about their treatments have not been fully considered. This is therefore a key focus of study in this paper.

Thirdly, literature in stroke, [27] brain injury [28] and older patients [29] has reported how patients, who have survived these illnesses and been left with a significant disability, when asked several months later, appear to have learnt to adapt to their situation (a process termed ‘response shift’) and therefore, report higher quality of life than anticipated. [30,31] In contrast when individuals who are well are asked about their preference for treatment which could leave them significantly disabled, they often report that they do not wish to receive such treatment: that is, they do not want to survive if they will be left significantly disabled. [32] Therefore, patients’ treatment preferences appear to be inconsistent. This so-called ‘disability paradox’ adds to the challenges associated with decision-making in acute stroke. [33] For doctors to be able to support patients in making treatment decisions, they need in-depth insight into each individual’s values, preferences and goals and how, and why these may change over time.

In this longitudinal qualitative study, we aimed to address several gaps in research involving acute stroke patients. Specifically, we explored how decisions were made regarding treatments in the context of an acute severe disabling stroke. To better understand this, we explored the early experiences of patients with a disabling stroke in hospital, their needs *[for information]* in the early period after a life-changing diagnosis and their views about surviving, potentially with significant disability. To gain deeper insight into their ongoing wishes and needs, at six months post-diagnosis, we explored their feelings about their situation *[being significantly disabled]* and views regarding information that would have been useful to them in the early period in hospital after their stroke.

While shared decision-making in acute stroke is a team approach, where various members of the multidisciplinary team (e.g. doctors, nurses, physiotherapists and dieticians) have input into patient management, the focus of our study is on decision-making regarding treatments that extended survival of the significantly disabled patient. These discussions are primarily conducted between the patient and their hospital doctor. Therefore, our recommendations are intended specifically for doctors taking care of acute severe stroke patients in hospital.

## Materials and methods

### Research design and methods

This study was informed by an epistemological position which recognises that illness experiences and, relatedly, treatment decision-making are socially and contextually informed. [34] This position informed our approach to both data collection and analysis. For instance, we developed topic guides (Table 1) which allowed us to explore what patients’ lives were like before suffering a stroke. We also explored their experiences soon after their admission in order to set the context for understanding the treatment decisions which were subsequently made.

**Table 1:** Guide to the topics covered in patient interviews.

Baseline	
Background	• The pre-stroke functional status of the patient: how they were managing at home before the stroke, any formal or informal care required, and their interactions with their family. • Reported preferences on surviving with significant disability: if they had made any advanced statements or had any thoughts of what they may want in terms of treatments if they had an illness that may result in them surviving with potentially significant disability.
Experiences	• Patients’ feelings about their situation post stroke: how they felt about their diagnosis and how they felt they were coping with the situation • Patients’ experiences in hospital in the early phase after the stroke: their interactions with staff
Needs	• Patients’ perception of information in general: whether this may help them understand their diagnosis, potential prognosis and help them make treatment decisions. • Their understanding about the goals of treatments that were being offered after acute stroke and what they would need to make decisions about these treatments
**Six month follow up**	
Experiences	• Patients’ thoughts and feelings having survived a physically disabling stroke, how they were managing on a day to day basis, their thoughts on their recovery process since hospital discharge
Needs	• back to their time in hospital, their thoughts on what (for example, information or support) could have been given to them in hospital which may have been useful to them.

Initial interviews took place within a week of a patient’s admission to the stroke unit. This time point was chosen to capture their early experiences after a disabling stroke. Also, we recognised that most treatment decisions that we were interested in exploring (such as initiation of enteral feeding) should have been taken within a week of admission.

Where possible, we undertook follow-up interviews at six months following first admission to the stroke unit. We chose this time frame because we recognised that, by six months, most patients would have plateaued with respect to their functional recovery.[35–37] Had we interviewed them at an earlier point in time (for example, at three months following first admission to the stroke unit), their recovery might have still been ongoing. By contrast, had we interviewed at a later point in time (for example, at one or two years following first admission to the stroke unit), this might have meant that other factors (for example, increasing age and frailty, recurrent stroke, declining cognition or death) might have adversely affected patients’ ability to be interviewed.

### Recruitment and data collection

We recruited adult patients who had been admitted to a stroke unit in a large teaching hospital in the United Kingdom. Our aim was to explore decision-making regarding stroke treatments that increased the likelihood that the patient who has been left with significant disability as a result of stroke will survive longer; to be eligible to participate, the stroke needed to have caused significant physical disability and the patient needed to have had decisions made regarding treatments (such as enteral feeding, parenteral fluids, antibiotics or intermittent pneumatic compression). Therefore, we recruited patients whose extent of disability was at least a modified Rankin scale score of four as a result of the stroke (i.e. they were unable to walk or attend to their own bodily needs without assistance).

The medical team (consultants, registrars and other trainee doctors who looked after the patients on a daily basis) identified eligible individuals who had the capacity to consent to participating in the study (i.e. the patient was able to understand and retain information and communicate decisions). If the patient’s speech or swallow was affected, the medical team assessed them to ensure this did not affect their decision-making capacity and that they were able to fully participate in an interview. In each case, before approaching the patient regarding in this study, the medical team also considered whether participating might cause distress.

Once the medical team determined that the patient was suitable to participate, they asked the patient for permission for the researcher (AV) to approach them and provide further information about the study. AV was a clinical doctor specialising in geriatric medicine who had previously worked in the stroke unit. However, patients were not informed about AV’s clinical background and they were advised that she would not be able to provide any treatment advice or medical information specific to their care.

AV then provided information about the study and obtained the patient’s formal written consent to participate. We made every effort to recruit patients of different ages, genders and ethnic backgrounds. However, we recognised that patients with an acute disabling stroke are a group who are hard to recruit: many patients are medically unwell and the impact of the diagnosis was noticeably distressing to some of them. Therefore, we made a pragmatic decision to interview all eligible individuals who had agreed to take part. As the clinical team did not keep a log, it is not possible to report the total numbers approached and, of those, how many declined to participate.

We endeavoured to recruit sufficient participants to address our study aims while not creating a dataset which would be too large and unwieldly to analyse in-depth. In practice, this meant that we stopped recruitment after on-going analysis of the interviews indicated that sufficient data had been obtained to address the study aims and that a point had been reached where patients were volunteering similar views. Hence we recognised that recruiting additional patients would not enhance the quality or diversity of the data collected.

Prior to the start of each interview, AV reassessed patients’ capacity to participate. This was done because we recognised that patients’ capacity might have changed from when they had initially been assessed as suitable to take part. Initial interviews were conducted in a private room in the stroke unit at a time convenient to the patient. Table 1 summarises the main areas explored. Where possible, we conducted six month follow-up interviews in the patient’s place of residence (i.e., either own home or care home). Telephone interviews were considered where the multidisciplinary team felt that it would not be safe for AV to visit the patient. Before contacting the patient to arrange the interviews, AV phoned their general practitioner to check the patient still had capacity to participate. This was part of the consent process at recruitment. Prior to follow-up interviews, AV reassessed the patient’s capacity. We summarize the main areas explored in these interviews in Table 1.

AV kept a reflexive diary. This detailed her interactions with the participants, including a record of why, based on what patients said in interviews, she had decided it would be insensitive or inappropriate to pursue certain lines of questioning. For instance, when AV explored patients’ feelings about potentially living with disability, only physical aspects of disability were discussed (for example, immobility and inability to perform activities of daily living). This was because recruited patients were assessed to have physical disability only, and exploring irrelevant disabilities (such as cognitive or intellectual) may cause the patient unnecessary worry. Also, as we will go onto describe, it became apparent to AV that, in the early stages post stroke, some patients did not appear to understand that the goals of some stroke treatments were to extend their life but they will be significantly disabled and that decisions needed to be taken regarding these treatments. Making this known to them either acutely or six months later could have caused undue distress.

Initial interviews took place between September 2017 and January 2018 and lasted 25 to 66 minutes. Six month interviews took place between April 2018 and July 2018 and lasted 32 to 56 minutes. All interviews were digitally recorded and transcribed in full with the patient’s consent.

### Data analysis

AV (who has received training in qualitative methods, including qualitative data analysis) and JL (a very experienced, non-clinical qualitative researcher) undertook data analysis. All data were analysed thematically using the method of cross-comparison.[38] This approach entailed repeated read through of all interviews to allow familiarization with the data (immersion). Interviews were then cross-compared to identify key findings which cut across different accounts (themes). Both inductive and deductive approaches were used; this allowed unanticipated themes to emerge from data as well as identification of material needed to address the study aims. Data were also analysed longitudinally to establish whether, and why, peoples’ needs and views changed over time. AV and JL analysed the data separately and wrote separate reports. They then met to discuss their interpretations, resolve any areas of disagreement (which were found to be minimal), and reach agreement on the main findings and themes. A coding frame was then developed which captured these findings and themes. Coded datasets were subjected to further analysis to allow development of more nuanced interpretations of the data and identification of illustrative quotations. Nvivo 11, a qualitative software package produced by QSR International, was used to facilitate data coding and retrieval.

### Ethical approval

This study was approved by Scotland A Research Ethics Committee (Ref: 17/SS/0029).

To safeguard participants’ confidentiality, pseudonyms are used.

## Results

Fifteen patients were interviewed within a week of their stroke. None were able to mobilise independently or wash and dress themselves without help after the stroke. Five also had speech impairment. The medical team assessed and confirmed that this did not affect their capacity to participate in interviews. Although we tried to include patients of varying backgrounds, most were of similar ages (70s) and ethnicities (White British).

At six months, thirteen patients had survived. Eleven had capacity to take part in an interview. All surviving patients had varying degrees of physical disability. Of these, two had ongoing speech problems but this did not affect their ability to participate in an interview. Eight of the eleven patients with capacity were living at home, two in care homes and one in hospital. Ten interviews took place in person. One interview took place over the phone due to safety concerns raised by the multidisciplinary team in hospital and the patient’s general practitioner.

Table 2 summarises the characteristics of our sample.

**Table 2:** Characteristics of study participants.

Patient characteristics	Number of participants (n = 15)
Mean age in years (range)	79 (53–93)
Female/Male	9/ 6
Independent prior to stroke	11
Formal care package prior to stroke	4
Do not Attempt Resuscitation order (in the first week)	3 in hospital, 2 from the community, 10 none
First stroke	15
Comorbidities (Charlson index: 0, 1–2, 3–4, > = 5) Max score = 33	Score 0(no comorbidities) = 6
	Score 1-2(mild comorbidities) = 5
	Score 3–4 (moderate comorbidities) = 3
	Score 5 and above (severe comorbidities) = 1
Type of stroke [39]	Total anterior circulation: 7
	Lacunar: 5
	Partial anterior circulation: 2
	Posterior: 1
**Post stroke**	
Modified Rankin score (mRs) (Scores 0–6) [40]	mRs 4^a^ = 4
	mRs 5^b^ = 11
Speech problem not affecting capacity or participation in interview	5
Fed enterally (Nasogastric or gastrostomy)	5
**At six months**	
Survived	13; 11 with capacity
Modified Rankin score (mRs) Scores 0–6 [40]	mRS 1 ^c^ = 1
	mRs 3^d^ = 7
	mRs 4 = 2
	mRs 5 = 3
	mRs 6 ^e^ = 2
Place of residence	At home = 8
	Nursing home = 4 (2 did not have capacity to be interviewed)
	Hospital = 1
Speech problem not affecting capacity or participation in interview	2
Fed enterally (nasogastric or gastrostomy)	0

^a^ Moderately severe disability; unable to walk or attend to own bodily needs without assistance

^b^ Severe disability; bedridden, incontinent and requiring constant nursing care and attention

^c^ No significant disability despite symptoms; able to carry out all usual activities

^d^ Moderate disability; requiring some help but able to walk without assistance

^e^ Dead

We found that patients’ pre-stroke functional status; specifically, whether they were largely independent or dependent on others for their activities of daily living (for example, showering and dressing), appeared to influence their experiences, views and involvement in making treatment decisions. Therefore, where appropriate, we have separated our reporting according to these two groups.

We begin by describing patients’ backgrounds and pre-stroke functional status. We then explore patients’ experiences and reactions to their diagnosis in hospital in the early period after a disabling stroke. We describe how patients’ need for hope appeared to influence their needs *[for information and support]* and views *[regarding treatments]* in the early period after a disabling stroke.

We then go onto consider how patients’ pre-stroke background and functional status appeared to affect their feelings about their situation six months later and how, and why, on reflection of their time in hospital, their needs *[for information]* may have changed based on the situation they now found themselves in.

### Pre-stroke background and early reactions to diagnosis of stroke

#### Patients who were independent pre-stroke

The majority of patients reported either living alone or with their family and described how they were constantly doing things for others. While this kept them busy, these patients also described how being part of a close network of family and friends gave them a sense of purpose. For instance, Dorothy, who was in her late 70s, described her role as a helper to her disabled friend: *‘I’m always helping other people*. *I’ve got…my friend…I usually drive her about because she’s in a wheelchair*. *‘(Dorothy)*.

Likewise, Edith, who was in her early 80s, described how she loved cooking and being involved in the lives of her grandchildren: *“I go away shopping an awful lot*. *And I make*, *like*, *pots of soup and I give it to my grandchildren*.*”*

These patients described how they had never considered a situation where they might be left disabled and how the stroke had come as a surprise to them: ‘*And life so far*, *never had any problem*. *It’s just come as such a surprise*, *‘cause I'm not a person to give in to anything*.*’ (Dorothy)*

The sudden loss of independence resulting from the stroke was described as devastating and as having given rise to a sense of loss, uselessness and profound apprehension relating to what other people would think about them in their disabled state. This included Colin, a man in his 70s who had lived alone and previously worked in healthcare. He described his feelings of anguish as he reported how he felt he had lost his dignity:

‘*Not walking*, *talking*. *I feel embarrassed when I meet people*. *They’ll say to me*, *look at the state of him*. *Well*, *there was today a girl came and washed me and it was the first time*. *It’s just that I’m not used to it*. *I feel absolutely terrible*. *I told her though*, *I’m so sorry* …*to see me in such a state*, *you know*, *all your private parts just hanging out and she’s washing it all…*.*I was just lying there and I was shutting my eyes hoping that she wouldn’t be long*. . . .*’ (Colin)*

However, many described how they had quickly transitioned from shock and distress to focusing on regaining their pre-stroke functional abilities. This included Edith who reported wanting to be able to do her own shopping again, ‘*I don’t want to get restricted from anything*’ and Larry, a man in his 50s, who reminisced about his independent past where he cared for his wife and children, and described wanting ‘*the use of my arm and leg again*’.

While continuing to focus on regaining their independence, these patients also reported that, should the circumstances arise where they were unable to independently care for themselves at home, they would accept formal or informal care. These patients suggested that they had family and friends to live for and, therefore, for them, survival, even with disability, seemed acceptable. For instance, Larry expressed his wish to ‘*be part of the family again so [my] wife don’t have to do everything*’. Similarly, Edith, who often helped her family by caring for her grandchildren, described how she felt she had so much left to live for, and also how she had people in her life who would care for her if necessary:

‘*I like to be amongst my family all the time*. *I can revolve round it (referring to disability)*. *Obviously if I find out I need a lot of help*, *I can get one of my granddaughters to stay with me and that*, *you know*.*’ (Edith)*

#### Patients who were dependent pre-stroke

These patients described prior restrictions to their mobility and ability to care for themselves, which had meant that they already had a formal care package which allowed them to live at home. In contrast to the group above, these patients described a general deterioration in their quality of life over the preceding years and also lacking a strong family network. In addition to a formal care package, some of these patients required help from friends and family with shopping and housework. This included Harriet, a woman in her late 80s, who was largely housebound. Harriet described how her nephew (her next of kin) visited her once a year and dealt with her finances and how she was reliant on the kindness of her neighbour who came in and helped her on a regular basis: ‘*Oh he [nephew] visits*, *yeh*, *he does the ‘big stuff’ you know*, *like me house*, *money and that but [name of neighbour deleted] she’s brilliant*, *she is*. *Does my shopping and all that*, *keeps an eye on me*”.

These patients reported how their pre-stroke illnesses had led to them leading very restricted lives. They also described how, while they had not been content with this situation, they had adapted to it. For example, Nigel, a man in his 70s, described what it was like to have a neurological condition which had meant that his life had been dictated by his illness:

‘*I don’t go out nearly as much*. *I don’t have as many circle of friends*. *I used to go out every six weeks to a lunch at [Place name removed]*. *I’ve had to curtail that*, *because my carers clash with the times*.*’ (Nigel)*

Due to their generally poor (and deteriorating) health, these patients reported multiple previous hospital admissions, where, on discharge, they had not returned to their previous level of functioning. Therefore, they described being mentally prepared for, and unsurprised by, a further deterioration in their abilities because of the stroke. Consequently, these patients, in contrast to those who were independent prior to the stroke, reported having thought about a situation where they might be left significantly disabled. In the event of this happening, Nigel, like others, described how he would not like his life to be prolonged:

‘*I don’t relish that idea to get spoon-fed……on a permanent basis*. *I hate the idea of being shut up in my own house*. *No*, *I would hate to be a vegetable*.*’ (Nigel)*

### Trying to cope with the diagnosis: Generating and sustaining hope

Regardless of their pre-stroke functional status, all patients described looking for hope of functional recovery. This included Nigel who described how: ‘*I am*, *I can and I bloody well will*: *where there’s breath*, *there’s hope*’ and Graham, a man in his 60s who, prior to the stroke, owned his own business, and enjoyed outdoor activities, who described how:

(*Re*: *recovery) ‘I’ll say [I am at] about 20% now*. *I can see the future higher up the ladder is there for me to climb up to it*.*’ (Graham)*

In-keeping with their wish to maintain a positive outlook, patients reported using different coping mechanisms to generate hope. Some reported comparing their functional state to that of other patients in hospital, and described how they thought that others were less fortunate than themselves as they had impairments that they felt were more severe. Such patients reported how this fuelled their hope that they may return to their pre-stroke state:

‘*When I was sitting in bed and watching the others*, *I kept thinking I’m lucky I’ve not got it as bad as they have*, *you know*. *I can swallow but I’ve just got to watch not to take a big mouthful*.*’ (Dorothy*, *a woman in her 70s*, *who was unable to mobilise or independently self-care post stroke)*

Others described how they valued speaking to stroke survivors they knew who had made full functional recovery and been discharged home. Indeed, such patients described seeking out stroke survivors, often with help from their family and friends, and welcoming hearing encouraging stories about their recovery:

‘*That was what she [friend] went through*. *I mean*, *I know that she’s okay*. *She’s a hundred per cent recovered but she was in a bad way*. *So that was good to understand that*, *just that there is light at the end of the tunnel*, *if you like*. *So that’s a positive experience*, *positive discussion I’ve had with someone who I know and I know she’s not lying to me*. *She’s looking good*. *She talks good*. *She’s not got any signs of a stroke*.*’ (Graham)*

Most patients also described how they needed information that was framed positively in order to help them maintain an optimistic outlook. In keeping with their need for hope, patients also described not wanting to engage with information that was ‘negative’; that is, which alluded to a lack of recovery or the possibility of living with significant disability. Many, like Graham, described even being prepared to accept information that was not necessarily correct, as long as it gave them hope:

‘*I want to know I’m going to get back to a hundred per cent; that’s what I believe inside that I’ll get back to*. *I think it’s vital to move forward*, *even if it’s…I was going to say even if it’s not completely true*, *but you’ve got to have a positive outlook*. *But if you come along and say*, *well you’ve got no chance*, *you’re going to be*, *you know*, *wrapped up in a wheelchair for the rest of your life*, *that’s not going to help me*.*’(Graham)*

Indeed, some described how they might lose trust in health professionals if given discouraging information. For example, Olivia, a woman in her early 80s recalled a situation where potentially dispiriting information would have been unhelpful:

‘*Well if somebody had said to my friend who died of the stroke*, *oh this is a very serious condition you’ve got yourself into*, *I don’t know if we’ll be able to get you out of this*, *well it wouldn’t have done him any good at all would it*? *I mean*, *where’s the trust that staff are helping*?*’(Olivia)*

### Treatment decision-making following diagnosis

It was against the above backdrop, that decisions about treatments such as intermittent pneumatic compression, enteral feeding, parenteral fluids and antibiotics were made.

#### Patients who were independent pre-stroke

Patients in this group generally reported taking all treatments that were on offer and, in their interviews, appeared to be unaware of the purposes of these treatments or even that treatment decisions had needed to be made. In keeping with their need for hope, many described assuming that treatments they were given were to help them make a full functional recovery. For example, Edith who was bed-bound after the stroke and needed a hoist to transfer from bed to chair described how she had been happy to take antibiotics for a urine infection because she assumed that these would help improve her mobility:

‘*Oh*, *I’ll take anything like that*. *Well*, *to get me on my feet*.*’ (Edith)*

Given their understanding of these treatments, some patients, including Graham, expressed their surprise that others might refuse them:

‘*I overheard someone very recently saying that if they’re not getting better they’d not want to be treated you know*, *they want to die*, *not me; I’m a fighter to the end*, *you know’*.*(Graham)*

#### Patients who were dependent pre-stroke

In contrast, these patients described having recalled the conversations with their doctors where treatment plans relevant to their situation had been discussed. They reported how they had informed their doctor that they would not want to have treatments that might leave them significantly disabled and therefore, when offered, they had declined these treatments.

This included Harriet, who declined enteral feeding as she felt that this might impact negatively on her already deteriorating health and quality of life:

‘*Oh gosh*, *no*. *That would be the last straw*. *I'd ask the Lord to take me away if that [referring to accepting enteral feeding] happened’*. *(Harriet)*

Similarly, Nigel, described how he had decided he did not want any treatments (referring to enteral feeding and intermittent pneumatic compression) that would increase the possibility of him being more dependent than he already was:

‘*I hate the idea of being shut up in my own house*, *no*. *I hate not knowing what is happening around me’ (Nigel)*

### Six month follow-up

#### Reactions to living with disability

At six months, none of the surviving patients had returned to their pre-stroke functional state and, since hospital discharge, many had experienced a further deterioration in their health and functional abilities.

#### Patients who were independent pre-stroke

Nearly three quarters of patients who were independent prior to the stroke required a formal care package to be able to manage at home at six months. This was a cause for despair for many such patients who described how, when they had left hospital, they had remained hopeful that they were going to be independent again. This included Brenda, a woman in her 80s, who shared her upset and grief at now needing carers to help her with daily care:

‘*Everyone kept saying*, *well*, *it’ll improve*, *but you don’t really realise how bad you’re going to be*. *I didn’t realise how much of a setback it was going to be*. *When people said they had a stroke*, *I didn’t realise what they were going through*, *whereas I certainly do now*.*’ (Brenda)*

Brenda, like many others who had been independent pre-stroke, reported a mixture of disbelief and frustration and described feeling unprepared for the reality of the situation she was now in:

‘*Very frustrating*, *because I can’t do what I normally do*, *like I went onto the computer yesterday to put in [husband’s name] prescription as I normally do*, *and I couldn’t press the bracket key*, *you know*, *the upper case*.’ *(Brenda)*

Indeed, it was evident from these patients’ accounts that their inability to do things they had previously taken for granted was a source of considerable grief. This included Irene, a woman in her 80s, who was previously independent and had regularly cared for her grandchildren. She now shared the anguish, embarrassment and distress she felt because of being unable to look after herself:

*‘I’m always embarrassed about asking somebody to help me do things that I feel I could be able to do myself*, *you know*. *For instance my left leg just now is sore because the way I’ve been lying I was* …*it’s over and I’ve got to get somebody to straighten it out for me*. *I can’t do that myself which I get quite annoyed about*.*’ (Irene)*

Some described how they were struggling to cope on a day-to-day basis and expressed their grief with their current situation:

‘*My walking and my balance is terrible*. *Well sometimes* …*I’ve fell a couple of times and my leg* …*got bruises on my other* …*while I was trying to collect things*.

*I’m just …how can I say …depressed. My words don’t come out right*. *My sentences don’t come out right.’ (Colin)*

Some patients also described having felt abandoned by staff once they were discharged from hospital. They noted how all therapy (specifically physiotherapy) had stopped following hospital discharge and suggested that if this therapy had been continued, they would not have been in the situation (disability requiring help from others) they were now in. This included Edith who described feeling alone and hopeless as a consequence:

‘*Oh*, *they’re finished with you once you’re out of the hospital*. *They don’t entertain you at all after that*. *To me they should carry it [referring to physiotherapy] on*.*’(Edith)*

This feeling of abandonment caused patients considerable upset. Edith, for instance, who had previously found meaning and purpose in her life by caring for her grandchildren now needed a hoist to transfer in the care home. Like others, she shared her feelings of despair and of not having anything left to look forward to in her life:

‘*I can go out when my sons come up if they’ve got…they take me outside in a wheelchair Some days they’re getting me in the car and I lower myself into it but I don’t ever get out of the car*, *nothing like that*. *They’ll open the windows and take me to the seaside and open the windows and let me look out and things like that but I feel it’s like a wasted life*. *You’re here for a wee while*, *the next place you go to that’s your dying off place that’s that*. *That’s what I feel*.*’ (Edith)*

Despite reporting feeling helpless and upset, some patients also described clinging onto hope of some functional recovery. This included Irene who recounted how she wished for an improvement in her balance:

‘*The physiotherapists were trying to get my balance sorted but it was hopeless*. *I kept falling back or to the side*. *So*, *unless I get that done there’s no way I can stand even*, *you know*. *I would like to improve on that*, *you know*. *Even just to stand up and get out of bed*, *just to move from here to the chair without having to use the stand aid*, *you know*.*’(Irene)*

A similar account was provided by Edith who described how she battled to cling onto hope and the possibility of some improvement:

“*Well*, *I can’t imagine myself being like this to the end*. *It’s not me*. *Oh*, *yes*, *definitely*. *They said apparently you can’t get any better but I did*. *I can get better*. *My brain’s fine and if I got help*, *through time I’d get this leg moving more because I’d like to be a wee bit more independent when it comes to the toilet and that if I can use…get on to one of these things that you push anyway*. *To be able to walk*, *maybe with a Zimmer*. *(Edith)*

#### Patients who were dependent pre-stroke

These patients, like those who were independent pre-stroke, described needing more help than before to be able to carry on with basic daily activities (such as showering, dressing and mobilising to the toilet) However, in contrast to those who were independent pre-stroke, it was evident from their accounts that this group of patients had given up battling for hope of further functional recovery. This included Nigel, who, six months previously, had already described a situation where he was largely housebound but had been able to mobilise independently (though slowly) indoors, was able to prepare a simple lunch and had got out (albeit only occasionally) to see his friends. Now, he described being fully housebound and needing help from his carers to get to the toilet and perform simple kitchen tasks. Despite this deterioration, he described, in a very ‘matter-of-fact’ manner how he:

‘*Would like just to have a more active life*, *If it (his mobility) comes*, *it comes*, *and if it doesn’t come*, *well that’s fair enough*.*’ (Nigel)*

Likewise, Harriet, described how, against a background of deteriorating health, she had previously been able to shower herself and entertain friends for afternoon tea but was now needing help to shower and was no longer able to invite her friends over. She reported that, while she wished to improve, she had accepted her situation:

‘I *have a minder who comes in; my only visitor really* … *She comes and gives me a shower in the morning*. *I’d like to do that you know…but I'm coping with all that*. *I've got a special seat in my bathroom*, *where I sit for the shower’*. *(Harriet*, *a woman in her late 80s)*

### Retrospective views regarding information in the early period after the stroke

When patients were asked to consider what would have been useful for to them to have known before they were discharged from hospital, all patients, regardless of their pre-stroke functional status, reported their wish to have been given information which would have helped them better understand and prepare for the situation they were now in. For instance, Irene, in her current state, was in a care home and required hoist for transfers and a catheter to manage urinary incontinence. She reported:

“*Well*, *I think we wanted more information*, *you know*. *Excuse me*. *Just to understand why we’re like this*, *you know*.*” (Irene)*

Likewise, Brenda who was now needing carers to help her mobilise to the toilet and to shower, described how she wished she had been given information on what her future may have looked like:

‘*An idea*. *You can’t precisely say when it’s going to happen*, *an idea of what image of what’s ahead*.*’ (Brenda)*

However, while these patients described their wish to have had information in hospital, they were not specific about the type and timing of this information.

## Interpretation

In this study involving patients who had an acute disabling stroke, we have highlighted how the functional status of patients prior to their stroke and their need for hope of functional recovery appeared to impact on their experiences, views and involvement in decision-making. First, patients who were functionally independent prior to their stroke were emotionally devastated in the early period post stroke and described feeling abandoned and struggling to find a sense of purpose six months later. They also appeared to be unaware that treatments in the early period post stroke might extend their life, but that they might be significantly disabled. These patients did not engage in treatment decision-making and took all treatments in the hope of functional recovery. In contrast, those who were dependent prior to the stroke were more stoic, had considered treatment implications and were more involved in treatment decision-making. They often chose not to have treatments that might prolong their already deteriorating health and poor quality of life. While they reported adapting to and accepting their increased need for care six months later, they were also saddened by their increased disability and social isolation.

Second, in the early stages post stroke, stroke survivors looked for various ways to cultivate and maintain hope that they would recover to their pre-stroke functional state. This included seeking positive information from doctors and other sources. At six months, many of the same patients (especially those who had been independent prior to the stroke) continued to be hopeful of improvement in their functional abilities. However, they also reported wishing they had been given realistic information in the early period after their stroke in order to prepare for the situation they were now faced with. Therefore, there appeared to be a mismatch between patients’ need to maintain hope of functional recovery and their retrospective wish to have had realistic information in the early period after their stroke. We refer to this as the “hope-information paradox”.

### Reactions to diagnosis- and the need for psychological support

Our study provides empirical support for recommendations provided by the Stroke Association [3] and Harrison et al [41], that stroke survivors may benefit from psychological support on discharge from hospital. Psychological support has been shown to be beneficial in patients with dementia [42], cancer [43] and myocardial infarction [44] because it reduces rates of depression, improves patients’ ability to cope with their situation, better optimises patients’ remaining abilities and improves their quality of life. This would be an important consideration for stroke patients, who, as we observed, had unmet psychological needs which seemed to contribute to a poor or suboptimal quality of life. In addition, our findings suggest that the type and amount of support required by patients who had suffered a disabling stroke may depend upon their functional status prior to the stroke and may also change over time. Therefore, different forms of psychological support (e.g. counselling[45], support groups [46], clinical psychology [47] and be-friending services[48]) may be appropriate for different patients at different time points. For example, those who had been functionally independent prior to the stroke may initially benefit from attending clinical psychology services and emotional support groups in order to come to terms with their diagnosis and loss of independence. Such patients would also benefit from be-friending services and peer support in the longer term to address social isolation as would patients who were functionally dependent prior to the stroke. A stepped care approach to psychological care after a stroke (that is, where patients are identified and treated and ‘stepped up’ to more intensive treatments based on their clinical need) was proposed by the National Institute of Health and Excellence in 2011 [49,50]., Although there are pathways into psychological services for stroke patients in the U.K, [41,51], patients in our study did not seem to have benefited from this. We recommend that the multidisciplinary team in hospital, in collaboration with specialist neuropsychology services if appropriate, assess the type and amount of psychological support each patient needs prior to hospital discharge and collaborate with community services to assess ongoing patient needs.

### Involving patients in making treatment decisions

Similar to acutely unwell patients in the intensive care unit [18], we also found that it was not possible to involve some patients in treatment decision-making in the early period after their stroke. This was especially the case for those who had been functionally independent prior to their stroke. As we have shown, such patients may not appreciate that, in the early stages post stroke, some treatments might extend their life, but with significant disability. They may also struggle to engage with health professionals in order to make treatment decisions in the early stages post-stroke. We have also highlighted how the emotional impact of a life-changing diagnosis and patients’ need for hope may not be conducive to them receiving and understanding realistic information, rationally weighing up the pros and cons of treatment and/or expressing their preferences for these treatments [16,18]–these all being key to effective shared decision-making as described early on in this paper. Trying to involve these patients in making collaborative decisions may cause unnecessary psychological distress. [52] By contrast, patients who were already significantly disabled before the stroke, like frail older patients with chronic progressive conditions, may be mentally prepared for further deteriorations in their health, have considered consequences of different treatment options and have often decided not to have treatments that may prolong an already unsatisfactory quality of life. [53–56] Hence these patients may be in a better position to be more engaged in the treatment decision-making process. We recommend that health professionals explore patients’ pre-stroke functional status and consider if and whether the emotional impact of diagnosis may prove it challenging to involve them in making treatment decisions.

### The hope- information paradox

The need for hope which we have reported in this paper is not exclusive to stroke patients: for cancer patients and older patients too, communication of hope by health professionals is said to help such patients adjust to their diagnosis and improve their welfare and quality of life. [21,57,58] Striking a balance between providing a patient hope that s/he will functionally recover while not providing false hope is challenging. [59] We report two further dilemmas for health professionals. First, shortly after their stroke, some patients volunteered that if their doctor were to provide them unfavourable information regarding the likelihood of their recovery at that point, they would lose confidence in their doctor, thereby detrimentally affecting the doctor-patient relationship. Second, when asked at six months to look back to the period shortly after their stroke, patients described wishing that their doctors had given them realistic information that could have prepared them better for their current (unfavourable) functional status.

While there are no easy answers with respect to resolving the “hope-information paradox”, one potential solution is to consider using existing cancer communication strategies in the stroke context. Potentially relevant cancer communication approaches include ‘Ask-Tell-Ask’ and ‘Hope for the Best, Plan for the Worst’ approaches. For example, under the ‘Ask-Tell-Ask’ approach, a doctor would communicate poor cancer prognosis, then respond to the patient’s emotions and finally transition to talking about next steps. Under ‘Hope For The Best, Plan For The Worst’ approach, a doctor would join with the patient in embracing their hopes while simultaneously asking them to explore a back-up plan based on their prognosis. This could help cultivate the patient’s hope by seeking to understand their diagnosis and prognosis and thereafter re-orient the patient’s care based on the patient’s goals and objectives. [59] Yet there are some challenges to directly adapting cancer communication strategies for using in the stroke context: the illness trajectories for cancer and stroke are different. [59] Specifically, relative to cancer, stroke is an acute medical condition, many treatment decisions need to be taken early, and the trajectory of the patient’s condition is difficult to predict.[60] By appreciating that stroke patients too, like other patients, may require different types of information at different points in their illness trajectory,[20,61] health professionals may consider reassessing stroke patients’ need for information at different points during their hospital admission.

While it is difficult to make concrete conclusions from our small study, we recommend doctors consider the following:

1. Exploring the social context and early experiences of patients to gain an understanding of their views and needs.
2. Being aware that patients may need psychological support, and that this best assessed prior to hospital discharge.
3. Being aware that, while guidelines exist, they may not apply to acute stroke patients: a shared decision-making approach may not always be appropriate.
4. Adapting strategies used in cancer when communicating hope but maintaining realism for patients who have had an acute disabling stroke.
5. Assessing the information needs of acute disabling stroke patients at various points during their hospital admission.

### Strengths and limitations

We were successful in engaging and interviewing a group of patients at a time when they are often ‘hard to reach’ and therefore excluded from research. Following up these patients at six months has given us important insight into their ongoing (and changing) needs for information and support. However, due to the impact of a disabling stroke on patients’ physical and psychological states, it sometimes proved challenging to probe and explore some of their experiences and views in-depth. Our sample size was relatively small and all participants were recruited from one tertiary teaching hospital. This reduced the diversity of our sample and hence, potentially, the transferability of the findings to other populations. [62] Patients in this study had capacity: our findings are therefore not applicable to those without mental capacity.

### Recommendations for further research

Future researchers could consider investigating the views and needs of stroke patients from different socio-economic and ethnic minority backgrounds who may have different information and support needs. Interviewing doctors who look after severe stroke patients could give us important insight into how, and why, doctors may have made decisions on behalf of the patient or what other factors they may have considered in the context of treatment decision-making.

## Conclusions

Survivors of an acute disabling stroke have unmet psychological needs which may contribute to a poor quality of life post stroke. These needs must be identified and addressed to help patients cope with their situation. A shared approach with respect to treatment decision-making may not always be possible or appropriate for patients who have had an acute disabling stroke, especially when they may be emotionally distressed and wishing to maintain a hopeful outlook. Health professionals should therefore exercise professional judgement when trying to involve patients in decision-making in the early period after a disabling stroke. The mismatch between patients’ ongoing need to maintain hope of functional recovery at six months, but retrospectively wishing they had been given realistic information in the early period after their stroke further adds to challenges of shared decision-making. In order to achieve a balance between maintaining hope while not providing false hope, communication strategies used in cancer may be adapted to the acute stroke setting. We also recommend reassessing the information needs of patients at different time points in hospital.
